# Three-Dimensional Reconstruction of Pelvicalyceal System of the Kidney Based on Native CT Images Are 1-Step Away from the Use of Contrast Agents

**DOI:** 10.5152/tud.2022.21329

**Published:** 2022-03-01

**Authors:** Bakhman Guliev, Ali Talyshinskii, Ilgar Akbarov, Vyacheslav Chukanov, Petr Vasilyev

**Affiliations:** 1 Department of Urology, North-Western State Medical University named after I. I. Mechnikov, Saint Petersburg, Russia; 2 Urology Center with robot-assisted surgery of the Mariinsky Hospital Saint Petersburg, Russia; 3 Department of Urology, Uro-Oncology, Robot-Assisted and Reconstructive Surgery, Faculty of Medicine and University Hospital Cologne, University of Cologne, Cologne, Germany; 4 Department of Urology, University Hospital Basel, Basel, Switzerland; 5 Peter the Great St. Petersburg Polytechnic University, Saint Petersburg, Russia

**Keywords:** Non-contrast computed tomography, pelvicalyceal system, renal colic, three-dimensional reconstruction

## Abstract

**Objective:**

To describe special algorithm for the semi-autonomous 3-dimensional reconstruction of the pelvicalyceal system based on native computed tomography images of patients with upper urinary tract obstruction.

**Materials and Methods:**

Fifty patients with renal colic fitting to inclusion criteria were enrolled. All patients underwent computed tomography urography to perform 3-dimensional reconstruction of the pelvicalyceal system on the affected size based on excretory phase representing “gold standard” and on native phase, which was performed via Medical Imaging Interaction Toolkit program updated with the described algorithm. Five urologists estimated their similarities and the potential use of non-contrast models for interventional planning. Contralateral non-distended pelvicalyceal system was reconstructed to evaluate the viability of the proposed technology in such cases. Surface areas of contrast and non-contrast models were compared. Distended pelvicalyceal system of 1 patient was used to reconstruct virtual endoscopic view. Obtained 3-dimensional non-contrast pelvicalyceal system models were analyzed by an engineer for suitability for 3-dimensional printing.

**Results:**

The average surface area of contrast and non-contrast models was 3513 and 3371 mm^2^, respectively (*P* = .0818). Non-contrast 3-dimensional reconstruction was possible with all distended pelvicalyceal systems and with 9 non-distended cases. Properties of non-contrast models were estimated as 4.3 out of 5. Obtained models were suitable for their intraluminal reconstruction and potential 3-dimensional printing.

**Conclusion:**

Described semi-autonomous approach allows for 3-dimensional reconstruction of dilated pelvicalyceal system based on non-contrast computed tomography images.

## Main Points

- Proposed semi-autonomous update of Medical Imaging Interaction Toolkit program software makes it possible to reconstruct 3-dimensional (3D) shapes of moderate-to-severe distended pelvicalyceal system (PCS) without contrast agents within 10 minutes
- Obtained 3D virtual models are suitable for both reconstruction of virtual endoscopic view and 3D printing
- Further studies are needed to develop the fully automated algorithm for the 3D reconstruction of PCS based on native computed tomography scans and to reveal appropriate morphometric properties of kidney collecting system for being visualized in such an approach.

## Introduction

Conventional computed tomography (CT) represents the most informative modality to define kidney stones’ localization, density, and sizes.^1^ However, it also has a significant disadvantage in the face of the impossibility of 3-dimensional (3D) visualization of kidney pelvicalyceal system (PCS). A thorough examination of its anatomy is necessary to determine the most appropriate surgical strategy. In addition, there is growing interest in using 3D reconstructions of PCS for their 3D printing,^2^ plasticine modeling,^3^ and reconstruction of the virtual endoscopic view,^4^ which positively affects the process of patients counseling, preoperative planning, and surgical performance. The detailed examination of PCS and the implementation of novelties mentioned above are possible when performing CT urography (CTU) with contrast agents, leading to increased radiation exposure.^5^

Moreover, their use is limited in the presence of chronic kidney disease or allergy to such agents,^6^ restricting the number of patients eligible for CTU. This discrepancy dictates the need to develop assistance for the 3D reconstruction of the kidney collecting system based on non-contrast CT images, which was thoroughly described by Sung et al^7^ However, their approach is based on manual PCS segmentation, representing a time-consumed process.

In the light of the above, the purpose of this study is to describe a unique algorithm for semi-autonomous PCS segmentation making its 3D reconstruction easier and more available.

## Materials and Methods

After institutional ethical committee approval from Mariinsky Hospital (MEK05577), 115 patients presenting to the emergency department with renal colic between November 2020 and March 2021 were selected. Further enrollment was based on the following inclusion criteria: the presence of stone along the upper urinary tract and distention of kidney cavity on ultrasound and kidneys, ureters, and urinary bladder imaging, aged 18-65 years, and glomerular filtration rate ≥ 60 mL/min/1.73 m^2^. As a result, 50 patients were enrolled in this study. After informed consent was obtained from all participants of the study, further examination included CTU to have both contrast and non-contrast images for the 3D reconstruction of PCS on the affected size. Also, contralateral PCS was reconstructed to evaluate the viability of the proposed algorithm for the cases with normal anatomy. All CT studies were performed with 64-slice CT with a 0.5 mm step (Somatom Definition AS, Siemens) with patients in supine. This CT is characterized by a high-frequency x-ray generator and water method for its cooling. Power rating and tube potential were set at 80 kW and 120 kVp, respectively. The standard CTU protocol was used with intravenous contrast medium administration, Iohexol (Omnipaque 350 mgI/mL, GE Healthcare, Dublin, Ireland), the rate and dose tailored to the patient’s body weight without diuretic admission. The total effective radiation dose of CTU was approximately 31.7 mSv in all cases. The standard protocol for CTU consists of 3 phases: the native phase, a nephrographic phase (scanned at 80-120 seconds delay), and an excretory phase (scanned at 10-15 minutes delay).

**Figure 1. A-C.**
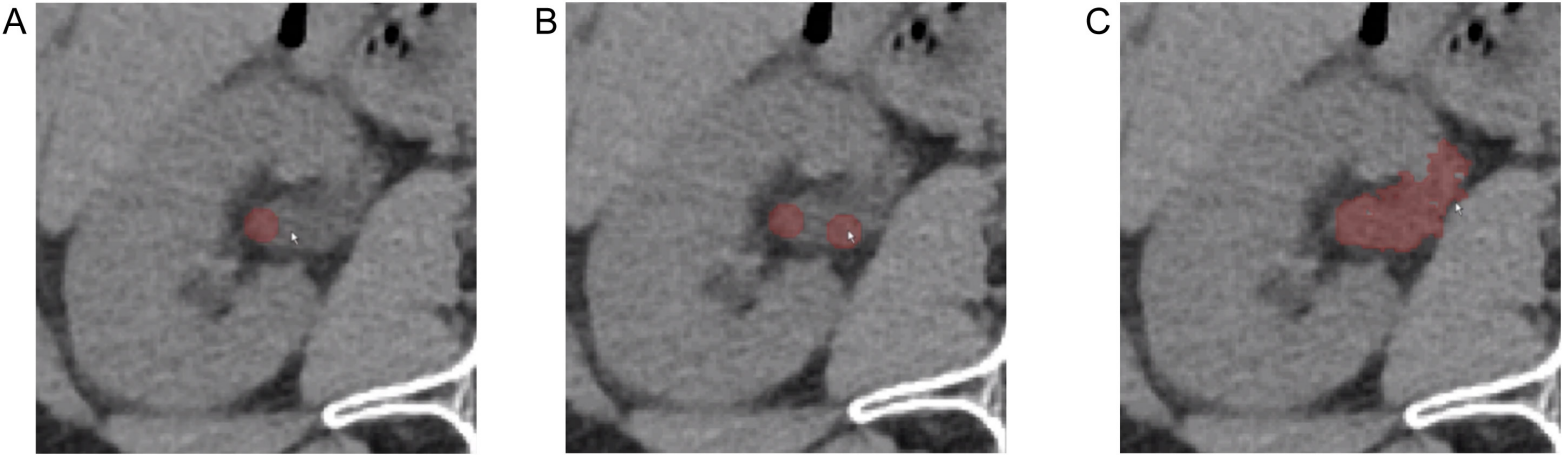
Circle footprints placed within PCS on the axial slice and the definition of PCS border. A, first-point position; B, second-point position; C, last-point position and proposed PCS border designation. PCS, pelvicalyceal system.

The 3D reconstruction process was performed via the Medical Imaging Interaction Toolkit program with the dedicated update created by co-authors of this study (V.C. and P.V.). Briefly, this algorithm produces a region of interest annotation based on small input footprints. The footprints could be simple “circle” labels, but we are using a smart brush to preserve boundaries and improve the precision of the inputs. Using just 3 input segments could be enough to annotate the region of interest. No pre-trained models are required once the method has a few parameters and automatically adjusts it using the footprints. The method allows real-time correction: new footprints can be added and the segmentation will be updated in real time (Figure 1). After the PCS border is defined on each slide of the axial plane, all slices are automatically fused to construct the final 3D model, which is polygonal due to the minimal difference between the urine density and adjacent structures being smoothed with the use of the same algorithm. After contrast and non-contrast models of each patient were obtained, their surface areas were compared via 3-Matic (Materialise, Leuven, Belgium). Non-contrast 3D models’ appropriateness was analyzed by a competent engineer concerning the suitability for 3D printing. Moreover, both contrast and non-contrast models were used to create virtual endoscopic views via InsKid mobile software, described previously.^4^ Finally, 5 urologists with a Likert-scale questionnaire compared non-contrast models with their contrast analog about similarity and the potential use of former in planning endourological interventions.

Statistical analysis was conducted using the Statistical Package for the Social Sciences software 22.0 (IBM Corp., Armonk, NY, USA). Shapiro–Wilk test was used to evaluate the data distribution. For continuous data, the mean and standard deviation were calculated. Student’s *t*-test or Mann–Whitney test was used for comparison depending upon the data normality. The significant difference was determined at the value *P* < .05.

## Results

**Figure 2. A-C.**
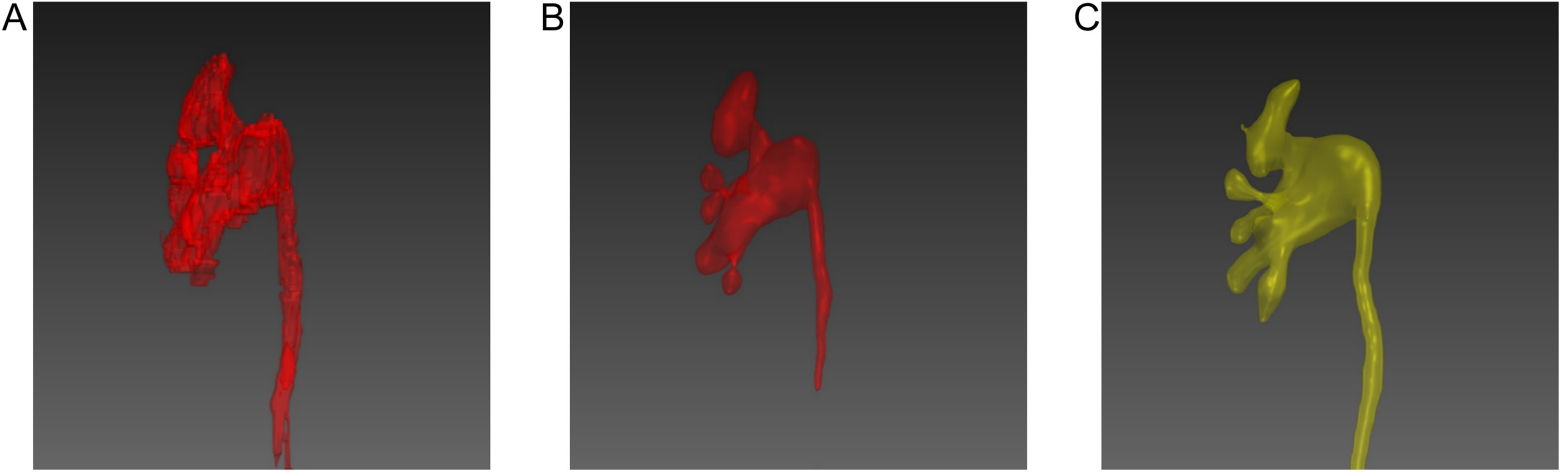
Anterior view of distended PCS being 3D reconstructed based on excretory and native phases. A, reconstruction of the non-contrast 3D model before smoothing; B, reconstruction of the non-contrast 3D model after smoothing; C, 3D reconstruction based on CT images of excretory phase. PCS, pelvicalyceal system, 3D, 3 dimensional; CT, computed tomography.

**Figure 3. A, B.**
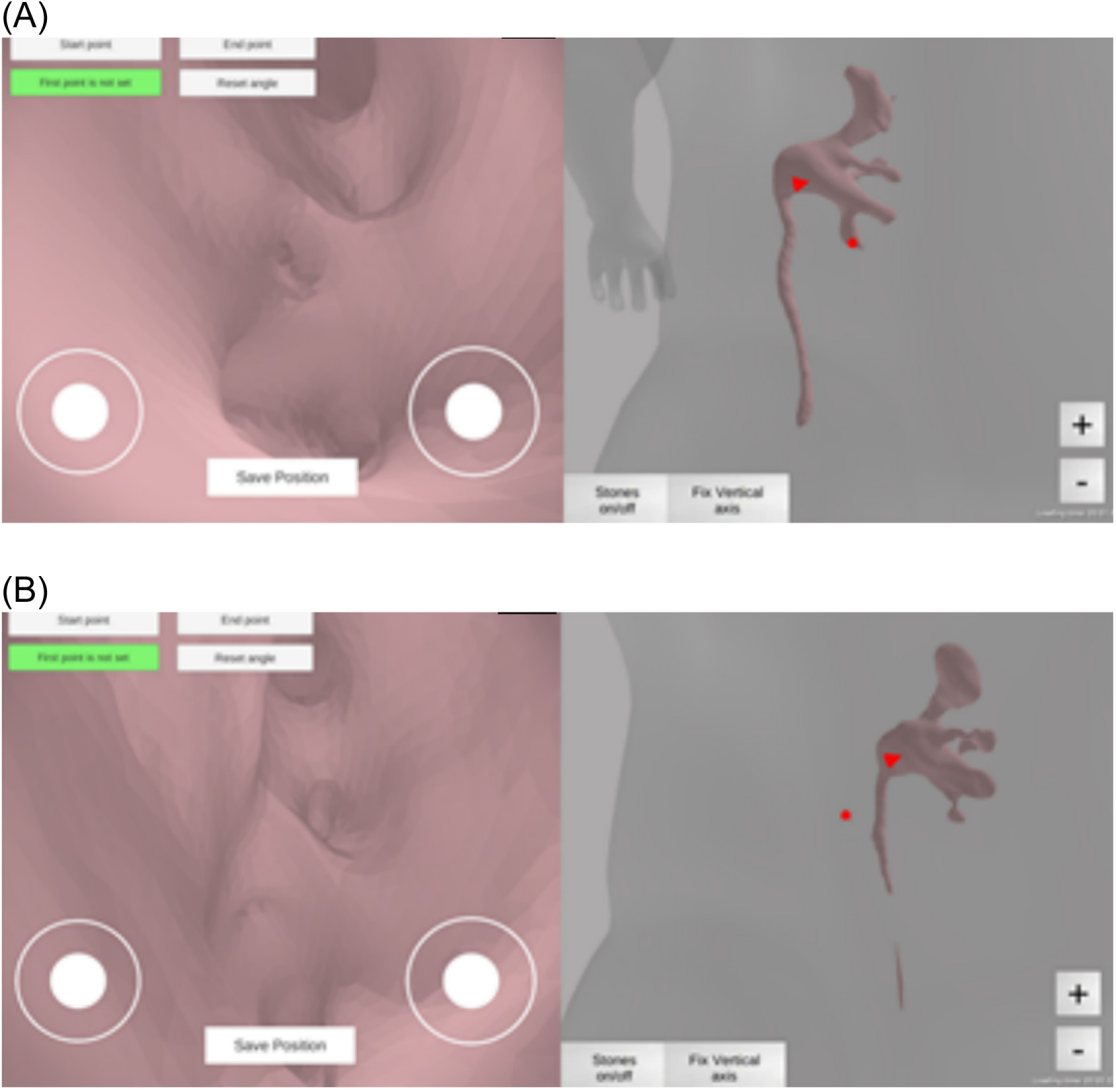
Reconstruction of the virtual endoscopic view based on contrast (A) and non-contrast (B) 3D models confirming the appropriateness of the latter for this purpose. 3D, 3 dimensional.

Patients’ demographics are shown in Table 1. The mean duration of 3D reconstruction of the affected PCS based on excretory and native phases was 17 ± 4 versus 490 ± 101 seconds, respectively (*P* < .0001), speaking in favor of the classic approach. However, the average surface area of contrast and non-contrast models was 3513 ± 420 and 3371 ± 387 mm^2^, respectively (*P* = .0818), confirming the almost identical reconstruction quality. According to the questionnaire result of 4.3 out of 5, the same opinion was shared by urologists, speaking for mathematical and visual similarity. Whole non-contrast 3D reconstruction was possible in all “dilated” cases (Figure 2) and 9 “non-dilated” cases, while 41 PCS on the non-affected side were not possible to be reconstructed based neither on contrast nor on non-contrast images. The qualities of the virtual intraluminal view of PCS were also similar, confirming the applicability of non-contrast 3D models for this purpose (Figure 3). After examination by an engineer, they were also confirmed to be appropriate for 3D printing. 

## Discussion

The conventional non-contrast CT provides urologists with reliable information regarding sizes, location, and the density of upper urinary tract stones. Also, it is possible to estimate different compartments of PCS based on 2-dimensional (2D) images, such as infundibulopelvic angle, and infundibular length and width, better preparing urologists before retrograde intrarenal surgery (RIRS) for lower calyceal stones.^8^ However, such data are insufficient for thorough examination of PCS anatomy compared to its 3D visualization, which enables urologists to save in mind the whole PCS anatomy of patients precisely during the intervention, increasing its effectiveness and reducing the frequency of associated complications.^9^

Most of the scientific evidence has shown that patient-specific preoperative planning based on 3D technology can improve peri- and postoperative parameters. So, Zhu et al^10^ explored the clinical value of 3D-image reconstruction technology on preoperative surgical planning and perioperative outcomes in laparoscopic pyeloplasty (LP) and concluded that it can provide accurate anatomical information and reliable guidance for preoperative operation planning and facilitates image-guided LP. Brehmer et al^11^ evaluated how 3D CT influenced the choice of access route and treatment outcome. Preoperative planning of complex stone situations with 3D CT significantly impacted operative procedure, resulting in a low number of access punctures. A similar study was conducted by Tan et al.^12^ exploring the potential benefits of 3D reconstruction technology in percutaneous nephrolithotripsy (PCNL) for complex renal calculi treatment. According to the results, there were significant improvements in the first-time puncture success rates and initial stone clearance rates 2 weeks after PCNL, confirming the role of the 3D reconstruction technology as an effective adjunct to PCNL in the complex renal calculi treatment. 

The ways to use 3D PCS shapes are not restricted solely with 2D images examination. So, Atalay et al^2^ described creating a physical model using 3D printing technology, which made it possible to improve the process of learning young residents. Gadzhiev et al^3^ described the process of recreating PCS by plasticine, which allows obtaining a physical model of the kidney collecting system within a short time to improve both planning and performance of PCNL. Another approach to 3D reconstructions is described in our previous paper^4^ that aimed to virtually reconstruct intraluminal PCS view via mobile software, improving patients counseling. The same software was used in this study when visually comparing contrast and non-contrast-based intraluminal reconstruction. 

Despite the diversity in the use of 3D virtual models, their reconstruction is cumbersome due to dependency on contrast agents, leading to increased radiation exposure on the patient. Moreover, patients with chronic kidney disease or allergy to the contrast agent do not fit CTU, leading to the applicability of the abovementioned ideas for treating relatively healthy patients. 

Three-dimensional reconstruction based on non-contrast images was described for structures with relatively constant shapes without branchings, such as heart,^13^ aorta,^14^ intracerebral bleeding,^15^ and kidney parenchyma.^16^ The kidney collecting system is difficult to reconstruct due to its variable and branching structure, a slight difference in density with the surrounding structures (vessels, fat, etc.), and the relatively “collapsed” normal state. The latter feature represents the main limitation for the use algorithms of 3D reconstruction of PCS based on non-contrast CT images. The same was stressed and thoroughly investigated by Sung et al.^7^ where the authors offer a protocol of intravenous infusion with diuretic load for artificial expansion of PCS before performing CT. According to their results, this approach leads to a significant increase in the surface area compared to the control group. The designation of the border of the PCS was performed manually on each slice, which is very time-consuming and limits the availability of this approach for routine clinical use. 

The classical procedure of 3D reconstruction of CT images consists of obtaining plain axial scans of the region of interest. The computer then provides a carefully selected “threshold” attenuation value. Each CT slice is scanned line by line and records each pixel’s exact coordinates that show an attenuation value higher than the chosen threshold. For example, if an attenuation value of +200 Hounsfield unit (HU) is chosen (optimal for the reconstruction of bone structures and urinary tract stones), only pixels with this attenuation value or more will be included in the 3D image. By lowering this threshold value, it is possible to reconstruct softer tissue. However, many tissues have a similar density and overlap when they are 3D.

A different approach is implemented in the described algorithm. With the help of input footprints in a circle shape, both the area of further reconstruction and its average density inside the mark are set simultaneously. If there is a stone, the density of PCS can be calculated at the mark boundary so that the stone remains harvested within the point and is not taken into account in the calculation. After the second point is set, the average density within the 2 labels is calculated, followed by automatic expansion of the reconstruction zone based on the HU difference. In our study, the density within the renal PCS varied between 0 and 15 HU, while the parenchyma density varied in the range of 30-45 HU. So, the boundaries of the PCS were determined with an increase in density >15 HU. Usually, 2-3 marks are enough to limit the area of interest. New footprints can be added for real-time corrects. 

All cases were reconstructed by the urologist (T.A.) after short instruction by the dedicated programmer, highlighting the simplicity of the approach and the absence of any learning curve. The average duration of PCS segmentation, its 3D reconstruction, and smoothing of the surface was 490 ± 101 seconds, which is optimal for implementing the described approach when planning either PCNL or RIRS.

This study also has several drawbacks. Although it was possible to reconstruct all distended PCS, only 9 non-dilated PCS allowed its reconstruction. The prime goal was to describe the technology, and we did not define appropriate morphometric parameters of PCS for its 3D reconstruction via described update. Reconstruction is semi-autonomic, which also requires human participation in its implementation. Finally, the described algorithm was not tested in the presence of kidney stone localized above ureteropelvic junction, which may theoretically distort the HU density difference between stone–urine–PCS border interaction. However, kidney stones up to 2 cm could be situated within a circle footprint to harvest them within the created PCS model. Subsequent investigations on this technology will be dedicated to solving the drawbacks mentioned above. The described update enables 3D reconstruction of the distended PCS based on native CT images of patients with upper urinary tract obstruction. 

## Figures and Tables

**Table 1.** Patients’ Demographics, Stone Properties, and Measurements of Models’ Surface Area

Parameter		Value
Male/female ratio		33/17
Age, year (mean ± SD)		51 ± 11
BMI, kg/m^2^ (mean ± SD)		27.8 ± ☐5
Glomerular filtration rate, mL/min/1.73 m^2^ (mean ± SD)		98 ± 16
Stone density, HU (mean ± SD)		720 ± 210
Stone size, mm (mean ± SD)		9 ± (7-11)
Stone location	UPJ	4
	Upper third of ureter	2
	Middle third of ureter	15
Lower third of ureter	29
Surface area of contrast models, mm^2^ (mean ± SD)		3513 ± 420
Surface area of non-contrast models, mm^2^ (mean ± SD)		3371 ± 387

SD, standard deviation; BMI, body mass index; HU, Hounsfield unit; UPJ, ureteropelvic junction.

## References

[1] Brisbane W Bailey MR Sorensen MD . An overview of kidney stone imaging techniques. Nat Rev Urol. 2016;13(11):654 662. 10.1038/nrurol.2016.154) PMC5443345 27578040

[2] Atalay HA Canat HL Ülker V Alkan İ Özkuvanci Ü Altunrende F . Impact of personalized three-dimensional (3D) printed pelvicalyceal system models on patient information in percutaneous nephrolithotripsy surgery: a pilot study. Int Braz J Urol. 2017;43(3):470 475. 10.1590/S1677-5538.IBJU.2016.0441) 28338309 PMC5462137

[3] Gadzhiev N Brovkin S Grigoryev V Tagirov N Korol V Petrov S . Sculpturing in urology, or how to make percutaneous nephrolithotomy easier. J Endourol. 2015;29(5):512 517. 10.1089/end.2014.0656) 25321395

[4] Talyshinskii A Guliev B Komyakov B Galfano A . Patient counseling through the pelvicalyceal-shaped labyrinth in search of an easy understanding of the upcoming stone removal: a pilot study. Urology. 2020;143:75 7 9. 32473936 10.1016/j.urology.2020.04.114 PMC7263277

[5] Brix G Lechel U Nekolla E Griebel J Becker C . Radiation protection issues in dynamic contrast-enhanced (perfusion) computed tomography. Eur J Radiol. 2015;84(12):2347 2358. 10.1016/j.ejrad.2014.11.011) 25480677

[6] Rudnick MR Leonberg-Yoo AK Litt HI Cohen RM Hilton S Reese PP . The Controversy of contrast-induced nephropathy With intravenous contrast: what is the risk? Am J Kidney Dis. 2020;75(1):105 113. 10.1053/j.ajkd.2019.05.022) 31473019

[7] Sung JM Jefferson FA Tapiero S et al. Evaluation of a diuresis enhanced noncontrast computed tomography for kidney stones protocol to maximize collecting system distention. J Endourol. 2020;34(3):255 261. 10.1089/end.2019.0719) 31984761

[8] Karim SS Hanna L Geraghty R Somani BK . Role of pelvicalyceal anatomy in the outcomes of retrograde intrarenal surgery (RIRS) for lower pole stones: outcomes with a systematic review of literature. Urolithiasis. 2020;48(3):263 270. 10.1007/s00240-019-01150-0) 31372691 PMC7220875

[9] Türk C Petřík A Sarica K et al. EAU guidelines on interventional treatment for urolithiasis. Eur Urol. 2016;69(3):475 482. 10.1016/j.eururo.2015.07.041) 26344917

[10] Zhu W Xiong S Xu C et al. Initial experiences with preoperative three-dimensional image reconstruction technology in laparoscopic pyeloplasty for ureteropelvic junction obstruction. Transl Androl Urol. 2021;10(11):4142 4151. 10.21037/tau-21-590) 34984180 PMC8661249

[11] Brehmer M Beckman MO Magnusson A . Three-dimensional computed tomography planning improves percutaneous stone surgery. Scand J Urol. 2014;48(3):316 323. 10.3109/21681805.2013.876552) 24521181

[12] Tan H Xie Y Zhang X Wang W Yuan H Lin C . Assessment of three-dimensional reconstruction in percutaneous nephrolithotomy for complex renal calculi treatment. Front Surg. 2021;8(8):701207. 10.3389/fsurg.2021.701207) 34746220 PMC8564007

[13] Shahzad R Bos D Budde RP et al.Automatic segmentation and quantification of the cardiac structures from non-contrast-enhanced cardiac CT scans. Phys Med Biol. 2017;62(9):3798 3813. 10.1088/1361-6560/aa63cb) 28248196

[14] Sedghi Gamechi Z Bons LR Giordano M et al. Automated 3D segmentation and diameter measurement of the thoracic aorta on non-contrast enhanced CT. Eur Radiol. 2019;29(9):4613 4623. 10.1007/s00330-018-5931-z) 30673817 PMC6682850

[15] Patel A Schreuder FHBM Klijn CJM et al. Intracerebral haemorrhage segmentation inNon-contrast CT. Sci Rep. 2019;9(1):17858. 10.1038/s41598-019-54491-6) 31780815 PMC6882855

[16] Khalifa F Soliman A Elmaghraby A Gimel'farb G El-Baz A . 3D KidneySegmentation from abdominal images using spatial-appearance models. Comput Math Methods Med. 2017;2017:9818506. 10.1155/2017/9818506) 28280519 PMC5322574

